# Stimulation of nitrogen removal in the rhizosphere of aquatic duckweed by root exudate components

**DOI:** 10.1007/s00425-013-1998-6

**Published:** 2013-11-24

**Authors:** Yufang Lu, Yingru Zhou, Satoshi Nakai, Masaaki Hosomi, Hailin Zhang, Herbert J. Kronzucker, Weiming Shi

**Affiliations:** 1 State Key Laboratory of Soil and Sustainable Agriculture, Institute of Soil Science, Chinese Academy of Sciences, Nanjing, 210008 China; 2 University of Chinese Academy of Sciences, Beijing, 100049 China; 3 Graduate School of Engineering, Hiroshima University, 1-4-1 Kagamiyama, Higashi-Hiroshima, Hiroshima, 739-8527 Japan; 4 Department of Chemical Engineering, Tokyo University of Agriculture and Technology, 2-24-16 Naka, Koganei, Tokyo, 184-8588 Japan; 5 Department of Plant and Soil Sciences, Oklahoma State University, Stillwater, OK 74078-6028 USA; 6 Department of Biological Sciences, University of Toronto, 1265 Military Trail, Toronto, Ontario M1C 1A4 Canada

**Keywords:** Fatty acid amides, Fatty acid methyl esters, Nitrogen-removal stimulants, Non-nutrient compounds, Duckweed, Root exudates

## Abstract

Plants can stimulate bacterial nitrogen (N) removal by secretion of root exudates that may serve as carbon sources as well as non-nutrient signals for denitrification. However, there is a lack of knowledge about the specific non-nutrient compounds involved in this stimulation. Here, we use a continuous root exudate-trapping system in two common aquatic duckweed species, *Spirodela polyrrhiza* (HZ1) and *Lemna minor* (WX3), under natural and aseptic conditions. An activity-guided bioassay using denitrifying bacterium *Pseudomonas fluorescens* showed that crude root exudates of the two species strongly enhanced the nitrogen-removal efficiency (NRE) of *P. fluorescens* (*P* < 0.05) under both conditions. Water-insoluble fractions (F) obtained under natural conditions stimulated NRE to a significant extent, promoting rates by about 30 %. Among acidic, neutral and basic fractions, a pronounced stimulatory effect was also observed for the neutral fractions from HZ1 and WX3 under both conditions, whereas the acidic fractions from WX3 displayed an inhibitory effect. Analysis of the active fractions using gas chromatography/mass spectrometry (GC/MS) revealed that duckweed released fatty acid methyl esters and fatty acid amides, specifically: methyl hexadecanoate, methyl (*Z*)-7-hexadecenoate, methyl dodecanoate, methyl-12-hydroxystearate, oleamide, and erucamide. Methyl (*Z*)-7-hexadecenoate and erucamide emerged as the effective N-removal stimulants (maximum stimulation of 25.9 and 33.4 %, respectively), while none of the other tested compounds showed stimulatory effects. These findings provide the first evidence for a function of fatty acid methyl esters and fatty acid amides in stimulating N removal of denitrifying bacteria, affording insight into the “crosstalk” between aquatic plants and bacteria in the rhizosphere.

## Introduction

Nutrients from non-point sources have become a major source of water contamination in many parts of the world. According to China’s first pollution survey, agriculture is responsible for 67 % of phosphorus (P) and 57 % of nitrogen (N) discharges (MEP et al. 2010), threatening water quality and human health (Beman et al. 2005; Shi et al. 2009). Excess N from agricultural non-point sources can also lead to changes in composition and diversity of ecosystems over extended time frames (Stevens et al. 2004; Isbell et al. 2013). One ecological approach used to treat polluted agricultural and industrial wastewater containing high nitrate is the use of plants with high nitrate-removal capabilities (Siddiqi et al. 1998) or the stimulation of bacterial denitrification in afflicted soils and water bodies (Mulvaney et al. 1997; Constantin and Fick 1997). Over the past several decades, aquatic plants have been increasingly adopted for this purpose (Tripathi et al. 1991; Li et al. 2009), in part because of the positive greenhouse impact of their use, and the relatively low cost involved.

Recently, we reported the acceleration of N removal by duckweed, a small floating aquatic plant within the family *Lemnaceae*, which is widely distributed in paddy fields all over the world (Zhou et al. 2010). It was shown that bacterial denitrification rather than plant uptake of nitrate was principally responsible for N removal (Bachand and Horne 2000; Zhou et al. 2010). However, the biochemical mediators underlying the enhancement of bacterial denitrification remained obscure. Several authors have suggested that plants may favor bacterial metabolism through root exudates in the rhizosphere (Mounier et al. 2004; Henry et al. 2008). However, most of the studies investigating the effect of root exudates on N removal have focused on the structure and activity of the soil-resident denitrifying communities (Baudoin et al. 2003; Henry et al. 2008; Shi et al. 2011) and much less on the plant component(s). Especially, little is known about the potential of aquatic plant root exudates to promote denitrification. In a recent study on *Spirodela polyrrhiza* (giant duckweed), a diverse range of phenolic compounds was found to contribute markedly to accelerated phenol degradation by bacteria in the rhizosphere (Toyama et al. 2009). By analogy, the release of compounds that may stimulate bacterial denitrification seemed plausible, and is therefore investigated here.

The importance of carbon for denitrification is very well known, and many studies have demonstrated the influence of plant-derived organic substrates on denitrification rates. Denitrification activity is positively correlated with soluble organic matter (Bijay-singh et al. 1988; McCarty and Bremner 1993) and with easily mineralizable carbon (McCarty and Bremner 1993). However, substrates described in such studies have only been shown to act nutritionally, as carbon sources for microbial growth, while possible signaling roles have not been explored. Moreover, previous studies have mainly focused on low-molecular weight compounds such as sugars, amino acids and organic acids (Paterson et al. 2007; Shi et al. 2011), with less emphasis on non-nutritional components, that may be responsible for chemical communication between plants and bacteria (Singer et al. 2003; Faure et al. 2009). Thus, classes of non-nutrient compounds that may lead to accelerated N removal remain unidentified.

*Pseudomonas fluorescens*, which is among the most common denitrifying microbes in natural soil and water environments (Gamble et al. 1977), was recently isolated from the duckweed rhizosphere where it acts as a naturally occurring aerobic denitrifying bacterium (Zhou et al. 2013). In the present study, two common duckweed species, *Spirodela polyrrhiza* (HZ1) and *Lemna minor* (WX3) were selected from the Tai Lake region of China, and the denitrifying bacterium *P. fluorescens* (ACCC 01047) was used to investigate the role of aquatic plant root exudates in enhancing N removal by denitrifying bacteria, under carbon-replete conditions, so as to exclude the possible contribution of root exudates as carbon-nutritional sources. We hypothesized that duckweed can secrete specific non-nutrient compounds that result in an increase of NRE of *P. fluorescens*. The main objectives of our study were: (1) to collect and isolate the root exudates of two aquatic species under natural and aseptic conditions, (2) to examine whether root exudates are an important factor in influencing N removal by denitrifying bacteria, (3) to identify the specific non-nutrient compounds, secreted from the roots of aquatic species, that facilitate denitrification, (4) to investigate their stimulatory effect on the NRE of *P. fluorescens*, and (5) to determine the origin of these compounds.

## Materials and methods

### Chemicals

Authentic standards of methyl hexadecanoate, methyl dodecanoate, methyl 12-hydroxystearate, oleamide, and erucamide were obtained from Sigma-Aldrich (St. Louis, MO, USA). Methyl (*Z*)-7-hexadecenoate was obtained from Cayman Chemical Co (Ann Arbor, MI, USA). The four fatty acid methyl esters and two fatty acid amides were dissolved in methanol before GC/MS analysis.

### Plant materials and bacterial strain

The duckweed species HZ1 and WX3 used in this investigation were collected from the paddy field drainage in the Tai Lake region of China. The two species were cultured in the laboratory in Steinberg nutrient medium for rapid frond multiplication and as the stock culture for subsequent experiments. The nutrient solution was changed twice a week to provide adequate nutrients and prevent algal growth during acclimation.

To obtain sterile duckweed cultures, 100 fronds were submerged in a 500-ml flask with 200 ml of 0.5 % (v/v) NaClO and were gently stirred for 30 min. These surface-sterilized plants were rinsed five times with sterile water, then aseptically transferred to 500-ml flasks containing 200 ml of sterile-modified Steinberg nutrient solution (100 mg/l MgSO_4_·7H_2_O, 98.9 mg/l Ca(NO_3_)_2_·4H_2_O, 12.5 mg/l NH_4_Cl, 1.76 mg/l KH_2_PO_4_, 0.18 mg/l ZnSO_4_·7H_2_O, 0.18 mg/l MnCl_2_·4H_2_O, 0.12 mg/l H_3_BO_3_, 0.04 mg/l NaMoO_4_·2H_2_O, 0.76 mg/l FeCl_3_·6H_2_O, 1.5 mg/l Na_2_EDTA·2H_2_O, pH 6.8), and maintained until use in the experiment. All plant materials were statically grown in an incubation room at 23 ± 1 °C under fluorescent lamps at 4,000 lux (16-h light and 8-h dark condition).

Duckweed rhizobacteria were collected using a modified method described by Yamaga et al. (2010). Ten duckweed fronds after enrichment were washed with sterilized water, and then transferred in a 1.5-ml microcentrifuge tube containing 1 ml of 5 mg l^−1^ sodium tripolyphosphate. The duckweed fronds were sonicated for six cycles of 5 s at 150 W to disperse surface-attached bacteria. The bacterial suspension was then added into a 250-ml flask containing 100 ml of sterile-modified Steinberg nutrient solution, supplemented with 65 mg l^−1^ glucose at 23 ± 1 °C with a day/night photoperiod of 16 h/8 h at 4,000 lux. After 3 days, cell-free supernatants were obtained by centrifugation of bacterial cultures at 12,000*g* for 15 min, and the supernatant fractions were filtered through 0.22-μm filters (millipore). The 100-ml cell-free supernatants were extracted with the same volume of dichloromethane (CH_2_Cl_2_). The organic phase was concentrated under vacuum on a rotary evaporator at 40 °C, and the residue was dissolved in 100 μl of methanol for further analysis.

The denitrifying bacterium *P. fluorescens* (strain ACCC 01047) was grown at 30 °C in a denitrifying medium (DM, 0.72 g/l KNO_3_, 1.0 g/l KH_2_PO_4_, 0.20 g/l MgSO_4_·7H_2_O, 2.8 g/l C_4_H_5_NaO_4_·6H_2_O, pH 7.0). Bacterial cells were cultured using 50-ml flasks with 20 ml of DM on an incubating shaker (120 rpm; 30 °C).

### Collection and separation of root exudates

**Fig. 1:**
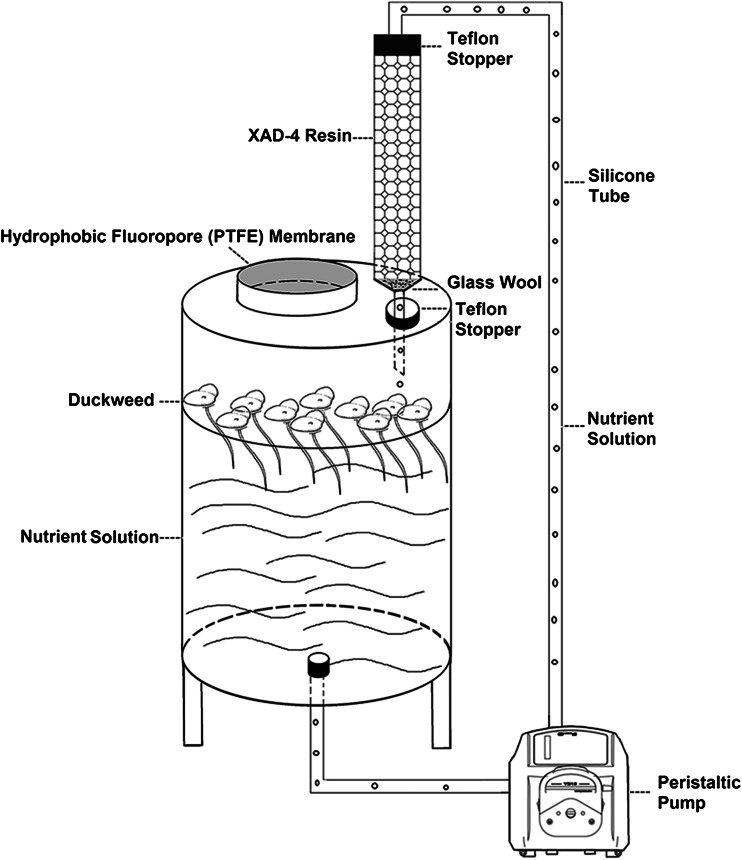
The continuous duckweed root exudate-trapping system

We used a modified continuous root exudate-trapping system (Tang and Young 1982) to collect root exudates from HZ1 and WX3 (Fig. 1). Under aseptic conditions, 140 cm^2^ (about 50 % coverage) of sterile duckweed frond culture was rinsed twice with sterile water and transplanted into the 4-l pot containing sterile-modified Steinberg nutrient solution. A hydrophobic fluoropore (PTFE) membrane was used under aseptic conditions to maintain a sterile environment. Under natural conditions, the duckweed fronds were rinsed just with distilled water, and the PTFE membrane was not used. A column (2 × 20 cm) packed with XAD-4 resin (Sigma) was connected to the top of the pot through a perforated Teflon stopper. The column was detached after 5 days, and eluted with 500-ml distilled water and then with 200-ml methanol. The methanol was evaporated under vacuum on a rotary evaporator at 40 °C.

**Fig. 2:**
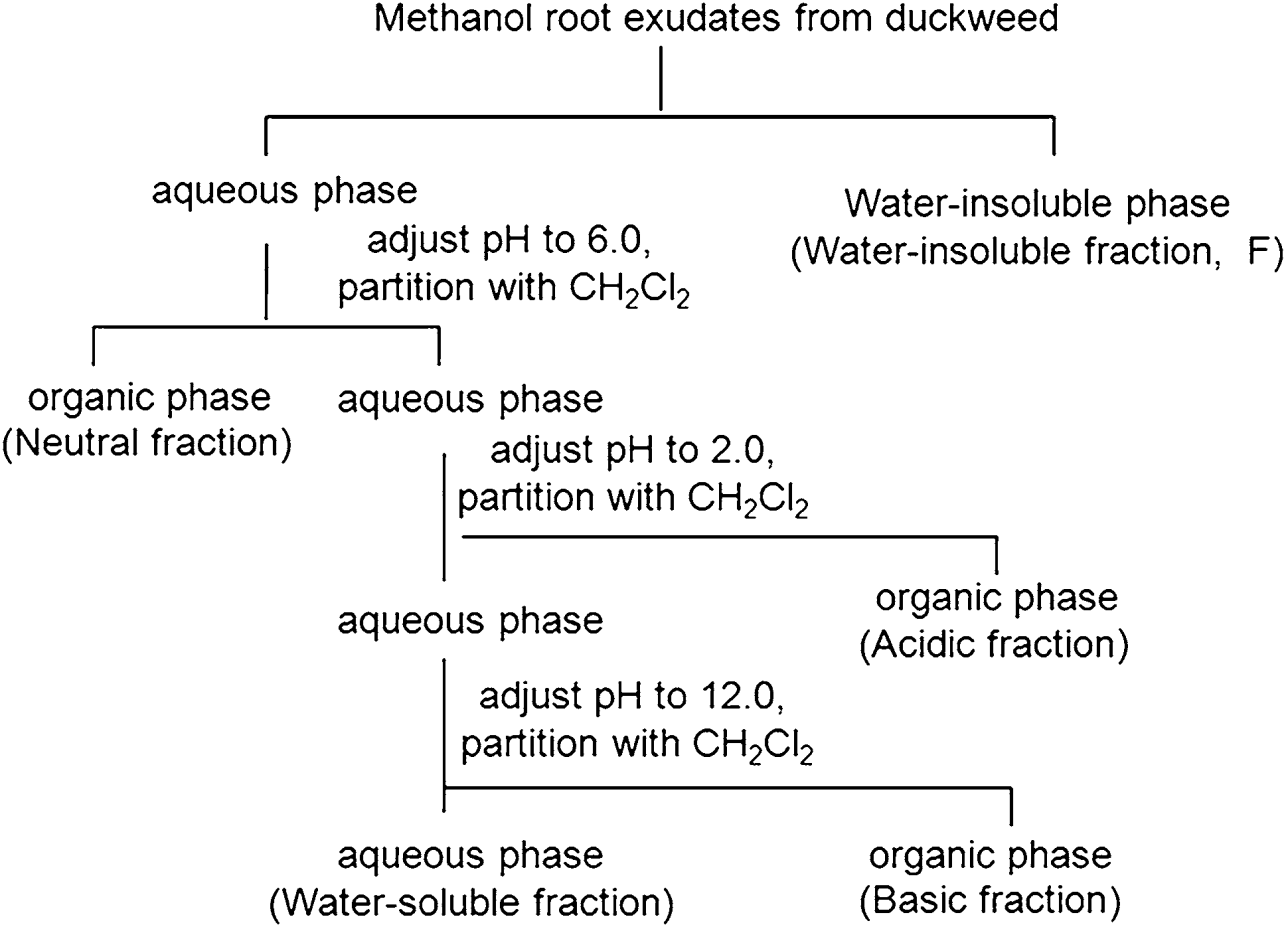
Fractionation process of root exudates from duckweed

The aqueous remainder was diluted with ultrapure water to 50 ml (pH 6.0) and subjected to the fractionation process shown in Fig. 2. The diluted 50-ml aqueous solution was first centrifuged (at 2,000*g* for 5 min, at 4 °C). The precipitate of the solution was defined as water-insoluble fraction, and the supernatant was then extracted three times with 100-ml CH_2_Cl_2_. The extracts (designated as neutral fraction) were combined, dried over anhydrous Na_2_SO_4_, concentrated under vacuum on a rotary evaporator at 40 °C, and dissolved in 2 ml of methanol. The acidic fraction was obtained in a similar manner by first acidifying the remaining aqueous fraction to pH 2.0 with 1 N HC1 and extracting with CH_2_Cl_2_. The basic fraction was obtained by adjusting the acidified residue to pH 12.0 with 1 N NaOH and extracting with CH_2_Cl_2_. Both fractions were concentrated to a final volume of 2 ml. The crude exudates and water-insoluble fractions (F) of the duckweed plant cultures were freeze-dried (Freezone Plus 2.5, Labconco, Kansas City, MO, USA), dissolved in 2 ml of methanol. All the fractions were stored in a freezer at −20 °C; aliquots of these samples (200 μl) were further concentrated using a jet of N_2_, dissolved in dichloromethane (CH_2_Cl_2_), and filtered through an autoclaved membrane filter (0.22 μm, millipore), for the bioassay.

### Bioassay

The bioassay used here was designed to avoid the potential for interference from carbon as a nutritional source, as follows: (1) sodium succinate (2.8 g/l) was added to maintain sufficient carbon for denitrification; (2) the total organic carbon of each fraction accounted for <2 % of that in DM. Bacterial cells from the late exponential phase, grown in Luria–Bertani medium (10 g/l tryptone, 5 g/l yeast extract, 10 g/l sodium chloride, pH 7.0), were recovered by centrifugation (at 5,000*g* for 15 min, at 4 °C) and resuspended in sterile DM (OD_600_ = 0.5). An aliquot (1 ml) of bacterial cells, and 200 μl of different fractions of duckweed root exudates, extracted with CH_2_Cl_2_, were added to a 50-ml flask with 19-ml DM, stirred and incubated on an incubating shaker (at 120 rpm and 30 °C). Control experiments were performed by substituting CH_2_Cl_2_ for fractions. As for the identified compounds, each was dissolved in CH_2_Cl_2_ and assayed in the same manner to ascertain whether they were active in N-removal stimulation. The assays were performed in triplicate. After a 72-h incubation period, 1 ml of the reaction mixture was centrifuged (at 10,000*g* for 10 min), and the supernatant was stored at −20 °C for later nitrate and nitrite analysis using an ion chromatograph (ICS-90, Dionex, Sunnyvale, CA, USA) and total N determination using ultraviolet spectrophotometric method after persulfate oxidation (APHA 1998).

The NRE and stimulation of each fraction were calculated as follows:

$${{\rm NRE}}\,(\% ) = \frac{{{{\rm TN_0}} - {{\rm TN_t}}}}{{{{\rm TN_0}}}}{{\rm \, \times \,100\,\% }}$$

where, TN_0_ is the initial total N concentration (mg/l), and TN_t_ is the total N concentration (mg/l) after 72 h.

$${{\rm Stimulation}}\,{{\rm of}}\,{{\rm NRE}}\,(\% ) = \frac{{{{\rm NRE}}\,{{\rm of}}\,{{\rm each}}\,{{\rm fraction}} - {{\rm NRE}}\,{{\rm of}}\,{{\rm CH_2Cl_2}}\,{{\rm control}}}}{{{{\rm NRE}}\,{{\rm of}}\,{{\rm CH_2Cl_2}}\,{{\rm control}}}}{{\rm \,\times \,100\,\% }}$$

### Identification of active fractions and rhizobacteria secretions

The chemical composition of active fractions and rhizobacteria secretions was analyzed by hyphenated gas chromatography/mass spectrometry (GC/MS). The GC/MS analysis was performed on a Hewlett-Packard 6890 gas chromatograph equipped with fused silica capillary columns HP-5MS (30 m × 0.25 mm, film thickness 0.25 μm) and coupled with a 5973 mass-selective detector. The injector was operated at 280 °C and the oven temperature was raised from 50 to 300 °C at a heating rate of 10 °C min^−1^. Helium at 1.0 ml min^−1^ was used as the carrier gas. One microliter of sample was injected in a pulsed splitless mode. The mass-selective detector was operated at an ionization energy of 70 eV, in the 35–650 amu range and with a scanning speed of 0.32 s. Compounds were identified by comparisons of retention time and mass spectra to commercial standards in the NIST libraries ver.2.0.

### Statistical analysis

Normality of data distribution (Shapiro–Wilk test) and homogeneity of variances (Levene test) assumptions were satisfied. Therefore, comparisons of effects of duckweed root exudates on the NRE were subjected to one-way ANOVA analysis of variance. The differences among duckweed root exudate treatments were evaluated by means of the LSD test at *P* < 0.05. All statistical analyses were performed by the PASW software package (version 18.0).

## Results

### Effect of crude duckweed root exudates on the nitrogen-removal efficiency (NRE)

**Fig. 3:**
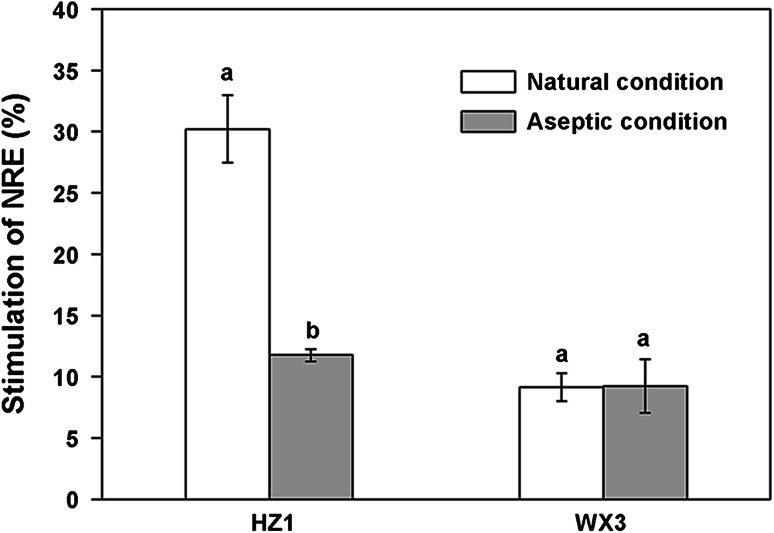
Stimulation of NRE of *P. fluorescens* (relative to the control) by crude root exudates from HZ1 and WX3 under natural and aseptic conditions. The NRE of the control was 34.65 ± 0.12 %. Values are mean ± SE from three replications. Different letters indicate significant differences at *P* < 0.05 by LSD test

During a 72-h incubation, the NRE amended with duckweed root exudates was higher than that of CH_2_Cl_2_ control and differed across treatments, with the exception of the acidic fractions of WX3 under the two conditions and the basic fraction of WX3 under aseptic conditions, where the efficiencies were lower than the control. Compared with the control, both the crude root exudates of the two duckweed species showed a significantly stimulatory effect (*P* < 0.05) on NRE, with the promoting rate of HZ1 under natural conditions significantly higher (30.21 %; *P* < 0.05) than under aseptic conditions (11.76 %), while no significant differences were found in WX3 under the two conditions (Fig. 3). This result indicates that duckweed root exudates are a positive factor in influencing N removal by denitrifying bacteria.

### Effect of different fractions of duckweed root exudates on NRE

**Fig. 4:**
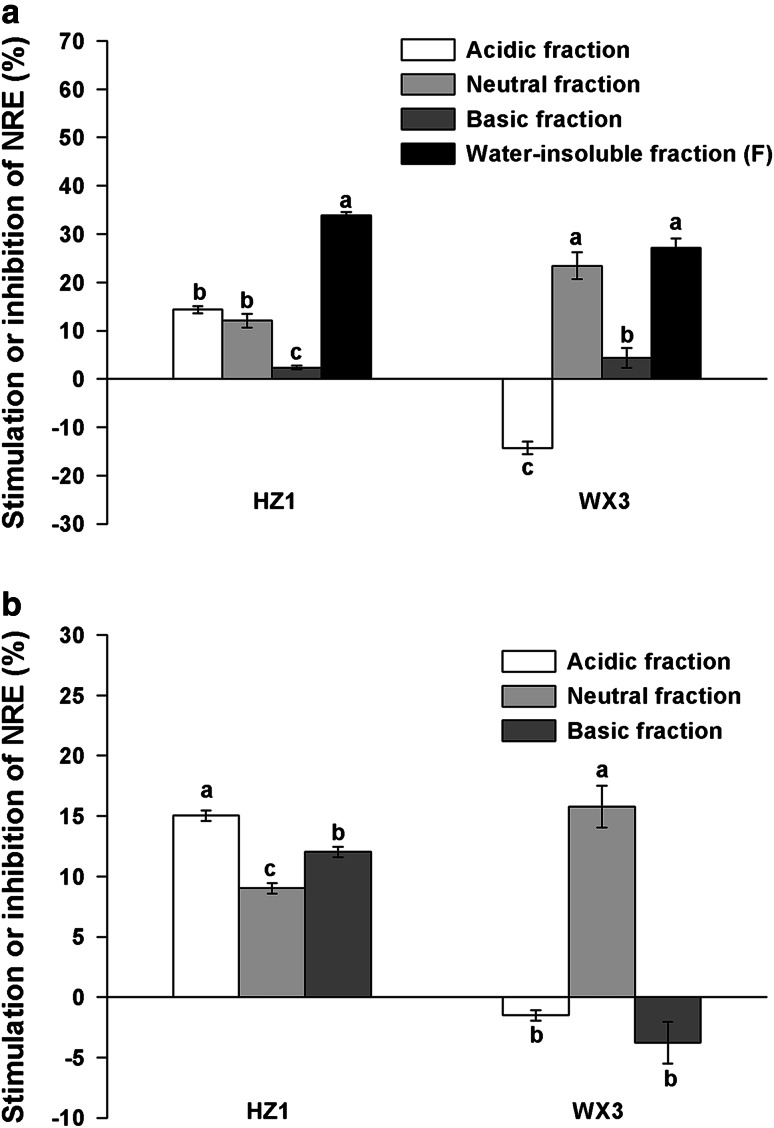
Stimulation or inhibition of NRE of *P. fluorescens* (relative to the control) by different fractions of root exudates from HZ1 and WX3 under natural (**a**) and aseptic conditions (**b**). The NRE of the control was 34.65 ± 0.12 %. Values are mean ± SE from three replications. Different letters indicate significant differences at *P* < 0.05 by LSD test

An effect of composition of root exudates from the two duckweed species on the NRE was observed. Under natural conditions, both the water-insoluble fractions (F) of the two duckweed species exhibited the most significant stimulatory effect (*P* < 0.05) on the NRE of *P. fluorescens* (Fig. 4a), and the NRE increased by 33.88 % with fraction F of HZ1, and 27.18 % with fraction F of WX3. The neutral fraction of WX3 and acidic fraction of HZ1 moderately enhanced NRE by 23.43 and 14.33 %. However, no stimulatory effect was found in the basic fractions of exudates of the two duckweed species. By contrast, the acidic fraction of WX3 had a 14.27 % inhibitory effect.

Similarly, the NRE of *P. fluorescens* also responded differently to fractions of duckweed root exudates under aseptic conditions. However, it displayed a weaker stimulation than that of the natural conditions. Among these fractions, the neutral fraction of WX3 and the acidic fraction of HZ1 stimulated the NRE most (*P* < 0.05) by 15.79 and 15.04 %, respectively, whereas the acidic fraction and basic fraction of WX3 even showed mild inhibitory activities (Fig. 4b). It needs to be mentioned that the amount of fraction F in sterile duckweed root exudates was very small, which makes it difficult to collect and identify this fraction.

Taken together, the higher overall stimulatory effect of crude root exudates under natural than aseptic conditions may result from the presence of fraction F, which showed the most significant stimulatory effect on the NRE of *P. fluorescens*. Furthermore, the neutral fractions showed a positive influence under all conditions in both duckweed species, indicating the possible presence of N-removal stimulants of duckweed origin in these fractions. These results led us to suspect that fraction F and the neutral fraction contained substances with strong stimulatory activities on N removal. Therefore, we focused on the bioactive compounds in these two fractions in our further GC/MS analysis.

### Identification of fraction F and the neutral fraction

**Fig. 5:**
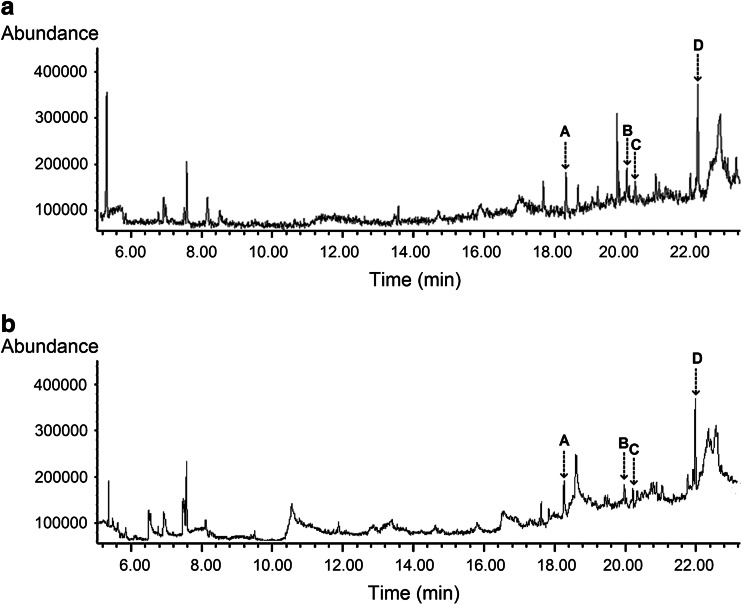
Ion chromatogram of the water-insoluble fractions from root exudates of HZ1 (**a**) and WX3 (**b**). **a** Methyl hexadecanoate (18.30 min), **b** methyl (*Z*)-7-hexadecenoate (20.04 min), **c** methyl dodecanoate (20.20 min) and **d** methyl 12-hydroxystearate (22.05 min)

**Fig. 6:**
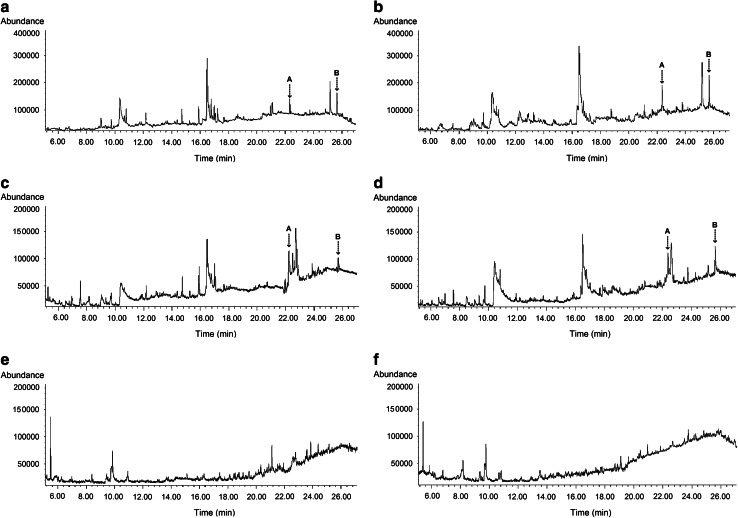
Ion chromatogram of the neutral fractions from root exudates of HZ1 (**a**, **c**) and WX3 (**b**, **d**) under natural and aseptic conditions, and the basic fractions of HZ1 (**e**) and WX3 (**f**) under natural conditions. **a** Oleamide (22.39 min) and **b** erucamide (25.67 min)

**Fig. 7:**
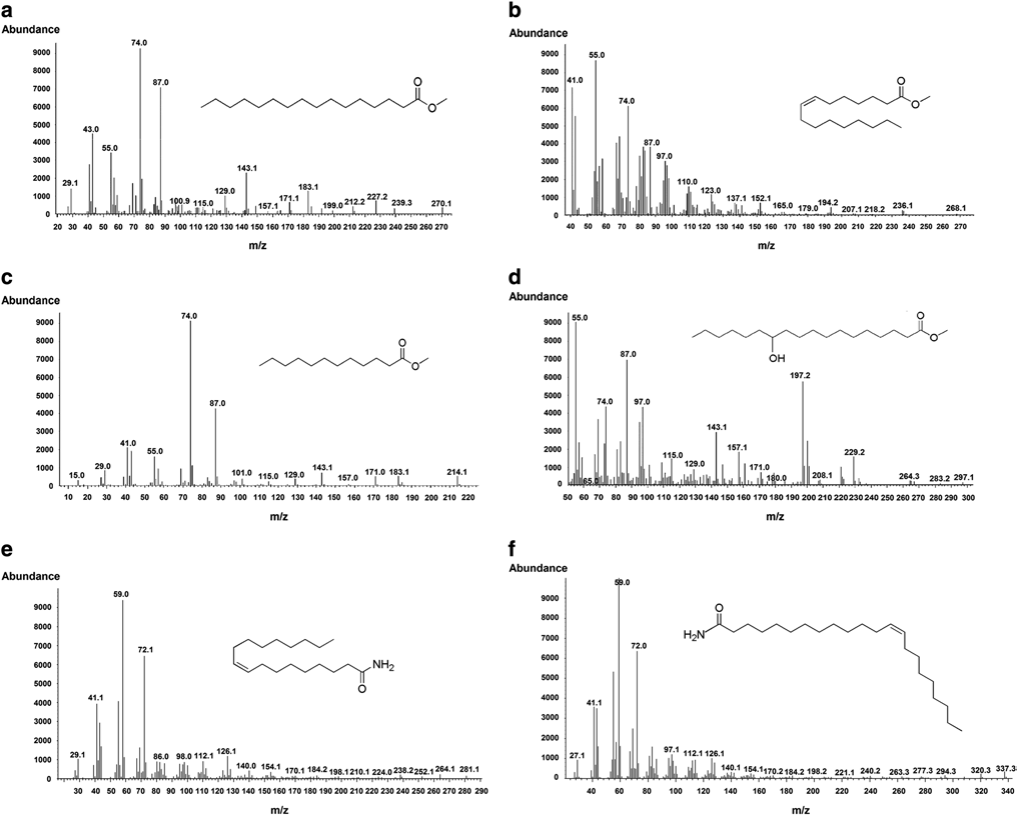
The mass spectra fragment patterns and chemical structures of the six identified compounds. **a** Methyl hexadecanoate, **b** methyl (*Z*)-7-hexadecenoate, **c** methyl dodecanoate, **d** methyl 12-hydroxystearate, **e** oleamide and **f** erucamide

Many peaks were observed in the ion chromatogram obtained by the GC/MS analysis of fraction F (Fig. 5). The mass-spectral patterns of the peaks were tentatively identified by comparing them with patterns archived in the US National Institute of Standards and Technology (NIST) mass-spectral library ver.2.0. Interestingly, four identical compounds were present in two water-insoluble fractions of duckweed root exudates (Fig. 5). The NIST mass-spectral library suggested the presence of methyl hexadecanoate (HDM, 18.30 min), methyl (*Z*)-7-hexadecenoate (cis-7-HDM, 20.04 min), methyl dodecanoate (DDM, 20.20 min), and methyl 12-hydroxystearate (12-HSM, 22.05 min) in fraction F. However, the four fatty acid methyl esters could not be found in the active neutral fractions. Two fatty acid amides, another chemical class of compounds, were identified as oleamide (22.39 min) and erucamide (25.67 min), under all conditions, and in both duckweed species (Fig. 6a–d). The mass-spectral patterns and structures of the six peaks are illustrated in Fig. 7. The identification of six compounds was further confirmed by comparisons with retention times to authentic standards. As a contrast, the basic fractions of HZ1 and WX3, which showed no effect on NRE under natural conditions, were also analyzed. However, the candidates were not present in these inactive fractions (Fig. 6e, f).

**Fig. 8:**
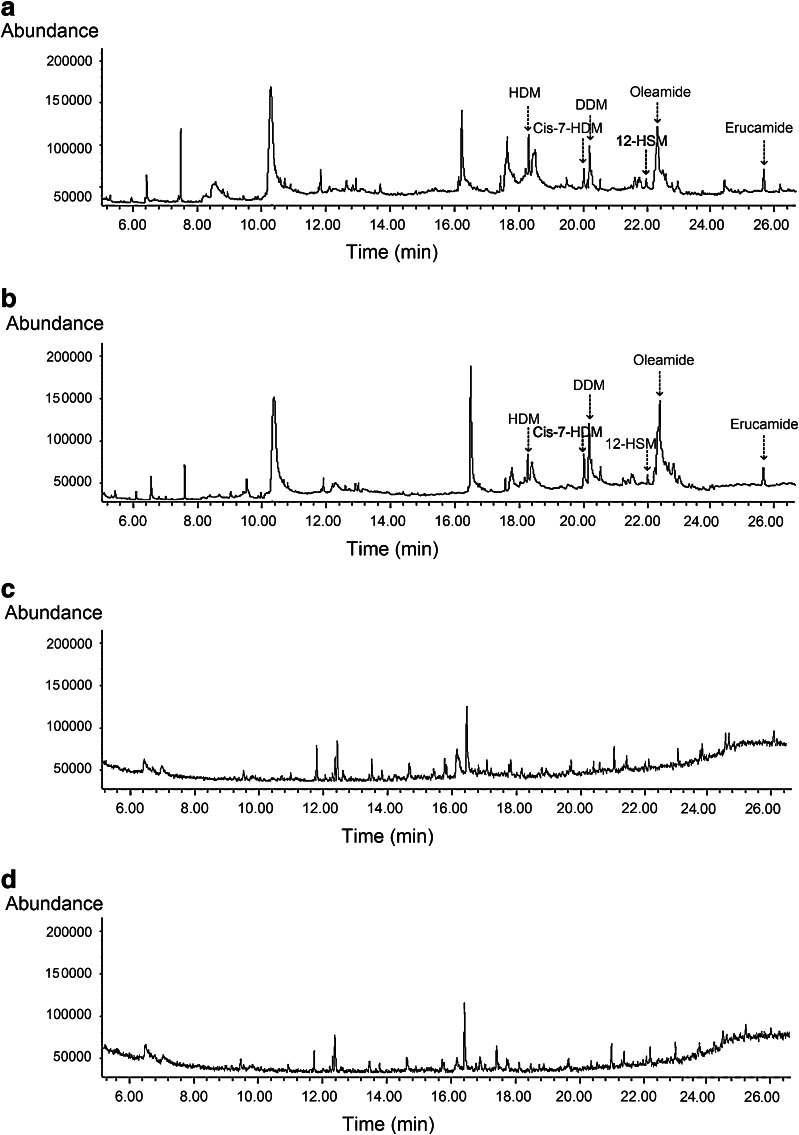
Ion chromatogram of the crude root exudates of HZ1 (**a**) and WX3 (**b**) under aseptic conditions, and secretions of mixed microbial populations isolated from the rhizosphere of HZ1 (**c**) and WX3 (**d**)

Finally, GC/MS analysis showed the presence of HDM, cis-7-HDM, DDM, 12-HSM, oleamide, and erucamide in the crude root exudates of the two duckweed species under aseptic conditions (Fig. 8a, b); however, they were not found in cultures of mixed microbial populations isolated from the duckweed rhizosphere (Fig. 8c, d). These results show that duckweed can release HDM, cis-7-HDM, DDM, 12-HSM, oleamide and erucamide.

### N-removal stimulatory effects of identified fatty acid methyl esters and fatty acid amides

**Fig. 9:**
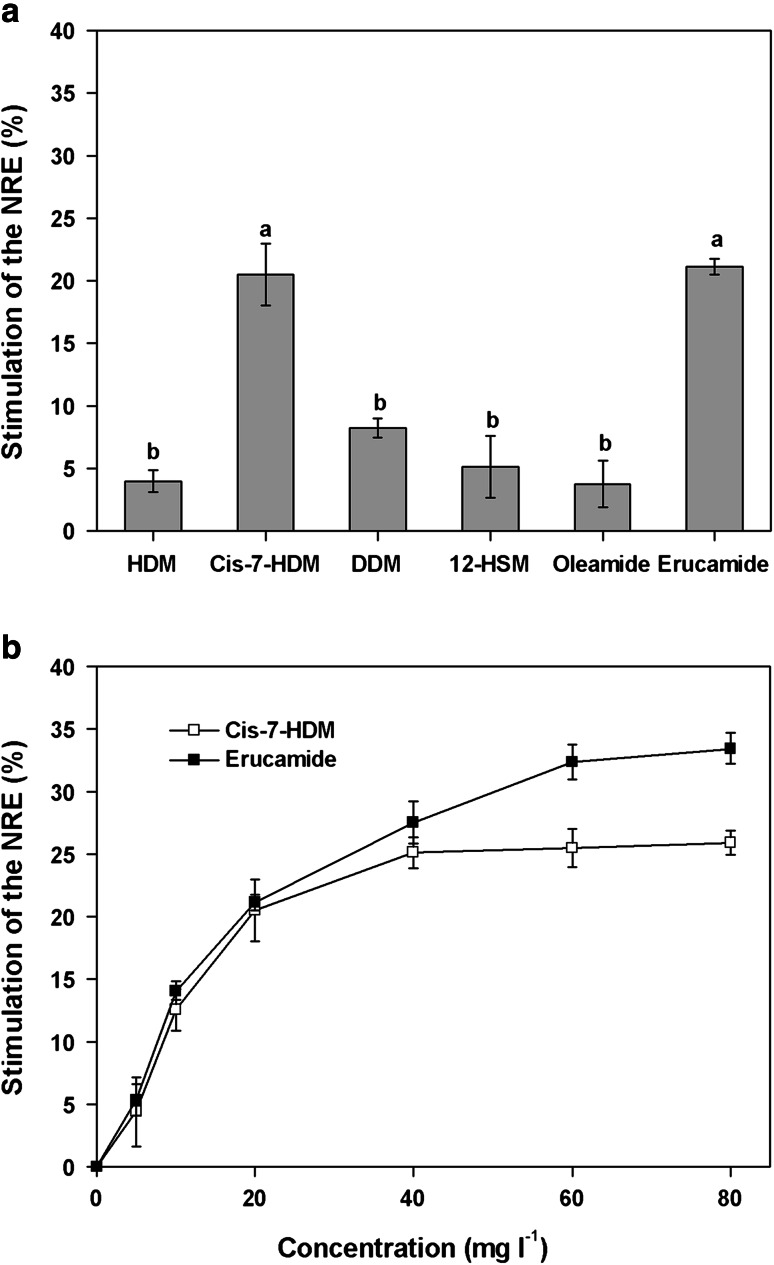
Stimulation of the NRE of *P. fluorescens* by six identified compounds at 20 mg l^−1^ (**a**) and effect of different concentrations of cis-7-HDM and erucamide on NRE in *P. fluorescens* (**b**). Values are mean ± SE from three replications. Different letters indicate significant differences at *P* < 0.05 by LSD test

Figure 9a shows the stimulatory effect of the identified compounds at 20 mg l^−1^ on NRE in *P. fluorescens*. HDM, DDM, 12-HSM and oleamide did not exhibit any significant effect, but cis-7-HDM and erucamide stimulated NRE in *P. fluorescens* by 20.51 and 21.13 %, respectively. These results clearly show that cis-7-HDM and erucamide, but not DM, DDM, 12-HSM or oleamide, can act as N-removal stimulants.

Figure 9b displays the dose–response relationships for cis-7-HDM and erucamide, showing that the stimulatory effect is concentration-dependent to some degree at concentrations below 80 mg l^−1^. The maximum stimulation of NRE by cis-7-HDM and erucamide was 25.9 and 33.4 %, respectively. Among the six identified fatty acid methyl esters and amides, erucamide demonstrated the strongest N-removal stimulation on *P. fluorescens*.

## Discussion

Denitrification is a major ecological process that removes N from ecosystems; it is a bacterially dominated respiratory process that reduces oxidized forms of N, returning them to the atmosphere as N_2_ gas, and occurs at greatly varying rates (Zumft 1997; Kirk and Kronzucker 2005). In soils and water bodies afflicted by excess NO_3_^−^, its stimulation, by targeting plant–microbial associations, has been used successfully to drastically reduce dissolved environmental N levels (Mulvaney et al. 1997; Constantin and Fick 1997; Bachand and Horne 2000). In several reports, transport of oxygen and secretion of physiologically active substances by plants have been linked to enhance microbial denitrifying activity in the rhizosphere (Mounier et al. 2004; Henry et al. 2008). Aquatic plant–microbial associations are furthermore attractive as cost-effective and environmentally friendly approaches to remediate aquatic environments contaminated with excess NO_3_^−^. However, no study hitherto has elucidated the role and composition of root exudates from aquatic plants in this context.

The present study focused on accelerated bacterial denitrification as a result of exposure to root exudates from two common aquatic duckweed species. A strong stimulatory effect of such exudates on the activity of a specific denitrifier (*P. fluorescens*) was observed, in general agreement with earlier studies (Lugtenberg et al. 1999; Bürgmann et al. 2005). Furthermore, we compared the effects and characterized the chemical components of duckweed root exudates collected under both aseptic and natural conditions. Such examinations are much needed to improve our still rudimentary understanding of the complex “crosstalk” involved in the stimulation of N removal by plants and bacteria in the rhizosphere. While some previous studies focused on the removal of heavy metals (Hou et al. 2007; Uysal and Taner 2009) or organic contaminants (Toyama et al. 2006; Kristanti et al. 2012) by duckweed-bacterial associations in aquatic environments, this is the first report describing the stimulation of N removal by duckweed root exudates.

We observed differential effects of various fractions of exudates from duckweed on NRE of *P. fluorescens* (Fig. 4). It is important to emphasize that experiments in this study were designed so that the contribution of root exudates to total organic carbon provision in each fraction (below 2 %) was negligible, and the carbon provided in the DM was sufficient to support the growth and denitrifying activity of *P. fluorescens*. Our results imply that substances in bioactive fractions of duckweed root exudates affect the activity of *P. fluorescens* as non-nutrient signals rather than by providing carbon nutrition. This observation is similar to the effect previously reported for artificial root exudates mimicking those of maize (Henry et al. 2008) and pine (*Pinus radiata*) where organic acids and not sugars were the compounds displaying activity (Shi et al. 2011). A small number of studies have also reported the effect of carbon substrates more generally on denitrification. Both glucose and starch have been shown to stimulate nitrous oxide reductase activity (Murray et al. 2004). Interestingly, the acidic fraction of WX3 under the two conditions examined, and the basic fraction of WX3 under aseptic conditions, displayed an inhibitory effect on NRE, which may explain the lower stimulatory effect of crude root exudates from WX3 than HZ1 (Fig. 3). Such negative effects may be the result of a direct, allelopathic, inhibition of *P. fluorescens* by certain compounds in the mixture. Although these compounds will not be favorable for N removal in polluted water bodies, they may have a significant role in inhibiting denitrification losses under field conditions, which is also frequently of importance, in particular to maintain the fertilizer-use efficiency of crops (Kirk and Kronzucker 2005; Subbarao et al. 2009; Magalhaes et al. 2012). Given the inconsistency in influence of the acidic fractions on N removal between the two species under variable conditions, the analysis of such specific fractions was not conducted in the present study.

When collecting exudates under natural (i.e., non-axenic) culture conditions, one must also consider that rhizosphere microorganisms, and not necessarily the plants themselves, may be the origin of some of the compounds collected. To test this possibility in the present study, HZ1 and WX3 were both sterilized to eliminate complicating microbial factors. Importantly, the stimulatory effect of crude root exudates on NRE of *P. fluorescens* was still observed under aseptic conditions. It is also interesting to note that the neutral fractions of HZ1 and WX3 all showed pronounced stimulatory effect under the two conditions. This consistency in effect implies that the compounds with N-removal stimulatory activity are indeed secreted from duckweed. Furthermore, direct GC/MS analysis of duckweed crude root exudates collected under aseptic conditions revealed the traces of six identified compounds, confirming that the potential N-removal stimulants are duckweed derived. A few other studies have also reported the ability of sterile duckweed to release bioactive substances (Baker and Farr 1987; Toyama et al. 2009). In view of the absence of six characterized candidates in cultures of mixed microbial populations from the duckweed rhizosphere, the possibility that rhizobacteria secrete these compounds can be essentially ruled out. Notwithstanding, the role of rhizosphere microorganisms cannot be ignored. Prikryl and Vancura (1980) found that wheat plants cultivated in the presence of *Pseudomonas putida* released up to twice the amount of exudates compared with axenically cultured variants. Therefore, it is not surprised that crude root exudates of HZ1 exhibited much stronger stimulatory effect on NRE under natural than aseptic conditions (Fig. 3). The main contribution of rhizobacteria from duckweed under natural conditions may be to induce the plant to produce more root exudates as N-removal stimulants, thus enhancing denitrification activity. Taken together, our data indicate that the origin of bioactive compounds involved in stimulating N removal is principally duckweed itself, and rhizobacteria may enhance this stimulation through beneficial interactions with duckweed to some extent.

Our current focus has been the analysis of the most bioactive fractions of duckweed root exudates. Two important classes of fatty acid derivatives were identified. In fraction F, fatty acid methyl esters were observed as the dominant chemical substances (Fig. 5), and two specific fatty acid amides were found in neutral fractions (Fig. 6a–d). However, these were not found in the basic fractions (Fig. 6e, f), pointing to the bioactive role of these compounds. It should be noted that other unidentified compounds in these fractions (both those represented in the gas chromatograms and those missing due to lake of volatility) may be also involved in signaling. However, the data presented here support the conclusion that cis-7-HDM and erucamide are the primary N-removal stimulants from duckweed root exudates. Compared with the identified saturated esters (HDM, DDM, 12-HSM), unsaturated cis-7-HDM has a specific carbon–carbon double bond at the 7, 8 position. As for fatty acid amides, the higher activity of erucamide than oleamide may be attributed to the greater length of the carbon chain and different double-bond positions. It is consistent with previous studies that the biological activity of lipids can be dramatically affected by the number/positions of double bonds or length of the carbon chain as they determine the structure, permeability, and affinity to lipophilic proteins, indeed lipophilicity in general (Jack-Hays et al. 1996; Krey et al. 1997; Boger et al. 1998). It is well known that in denitrifiers, the denitrification enzymes and related electron carrier system are located in the periplasm or the inner membrane (Zumft 1997). Cis-7-HDM and erucamide may have suitable structures and polarities for penetrating the bacterial cell.

Fatty acid methyl esters are widely distributed in aquatic and terrestrial environments, pointing to the possibility that fatty acid methyl esters may affect the activity of denitrifiers. They are found in root exudates of cotton (Li et al. 2013) and eggplant (Liu et al. 2009), in shoots of *Brachiaria humidicola* (Subbarao et al. 2008), and roots of *Rhazya stricta* (Atta-Ur-Rahman et al. 2008), as plant secondary metabolites, and several have been shown to exhibit considerable (mostly inhibitory) effects against bacteria and fungi (Nobmann et al. 2009; Huang et al. 2010; Gołębiowski et al. 2013). However, cis-7-HDM is rare and is only found in the sex pheromone of *Trogoderma glabrum* (Yarger et al. 1975), eliciting both arousal and attempted copulation (Greenblatt et al. 1976). Little is known about its biological activity in plants or bacteria. Fatty acid amides, such as oleamide and erucamide, are active as bioregulators within and outside the central nervous system in higher animals, acting as sleep-inducing (oleamide) or angiogenic (blood vessel building) factors (erucamide) (Wakamatsu et al. 1990; Huidobro-Toro and Harris 1996). Recently, erucamide was identified in human meibomian gland secretions (Nichols et al. 2007), and was reported to inhibit intestinal diarrhea and to regulate fluid volumes in other organs (Hamberger and Stenhagen 2003). Aside from mammalian systems, erucamide has also been characterized as the major toxic component in a golden alga (*Prymnesium parvum*), displaying cytotoxic activity, in particular on fish (Bertin et al. 2012), and also occur in extracts of pitcher plants of the genus *Heliamphora* (Jaffe et al. 1995).

To our knowledge, this is the first time that specific N-removal stimulants have been isolated and identified from the root exudates of aquatic fronds (*Spirodela polyrrhiza* and *Lemna minor*); moreover, no previous studies have examined the N-removal stimulatory activity of cis-7-HDM and erucamide. Watson et al. (2009) found that erucamide can stimulate cyclic AMP (cAMP) production in cells expressing a membrane-bound G-protein-coupled receptor (GPCR), known to be activated by fatty acid ligands. It is well known that DNR (dissimilatory nitrate respiration regulator) is a key activator of denitrification, belonging to the CRP (cAMP receptor protein)-FNR (fumarate and nitrate reductase regulatory protein) superfamily of bacterial transcription factors (Zumft 1997; Giardina et al. 2011). It is not unreasonable to postulate the specific role of erucamide in expression of genes involved in denitrification. Interestingly, erucamide was found to be one of the specific ligands of periplasmic HP-YceI protein involved in adaptation to acidic environments due to a linear chain of 22 carbon atoms in the bacterium *Helicobacter pylori* (Sisinni et al. 2010). Along with the ligand-binding properties of denitrification-implicated nitrite reductase and nitric oxide reductase (Sutherland et al. 1986; Collman et al. 2006), we might also postulate the role of erucamide in key denitrification enzymes in denitrifying bacteria. However, to support the hypothesis, the mechanisms of N-removal stimulants at the cellular and biochemical levels will need to be characterized in future studies.

As a fast-growing and widely distributed aquatic plant in nature, the production of duckweed is estimated at 10–50 t ha^−1^ year^−1^ dry biomass (Gijzen and Khondker 1997). Thus, substantial amounts of N-removal stimulants (i.e., cis-7-HDM and erucamide), which are environmentally friendly and functionally effective, are added annually to the water body during the process of root secretion, and this could have important additive effects over time in influencing the bacterial denitrification of nitrate-contaminated water in agricultural ecosystems. This is perhaps one of the main reasons for the observed low nitrate concentration in water bodies where duckweed is grown.

In the present study, we have presented a first glance at the biochemical mediators contained in root exudates from two duckweed species that underlie accelerated bacterial removal of N by denitrification. Our results demonstrate that, in the presence and absence of rhizospheric microorganisms, duckweed can secrete a fatty acid methyl ester (cis-7-HDM) and a fatty acid amide (erucamide) that display strong stimulatory effects on denitrification. Moreover, our findings suggest that N-removal stimulants exuded from duckweed might function as biochemical signals rather than carbon skeletons. Given the importance of developing cost-effective and environmentally friendly means to remove excess N from water bodies, the present findings represent a significant step forward in our understanding of the interaction of aquatic plant roots and microbes in the rhizosphere and outline the potential for future rhizosphere manipulation in the context of denitrification in ecosystems.

## Abbreviations

HZ1: *Spirodela polyrrhiza*
WX3: *Lemna minor*
N: Nitrogen
NRE: Nitrogen-removal efficiency
GC/MS: Gas chromatography/mass spectrometry
F: Water-insoluble fractions
HDM: Methyl hexadecanoate
Cis-7-HDM: Methyl (*Z*)-7-hexadecenoate
DDM: Methyl dodecanoate
12-HSM: Methyl 12-hydroxystearate
